# Comparison of Open-Book and Closed-Book Examinations and Nursing Students’ Perceptions of Open-Book Examination

**DOI:** 10.7759/cureus.100925

**Published:** 2026-01-06

**Authors:** Hepsi Bai Joseph, Asha P Shetty, Geetarani Nayak, Bhanuprava Mallick, Sandhiya K., N. Sadhana Priyadarshini, Manjula Kumari Nayak

**Affiliations:** 1 Pediatric Nursing, All India Institute of Medical Sciences, Bhubaneswar, Bhubaneswar, IND

**Keywords:** closed book exam, comparison, nursing students, open book exam, perception

## Abstract

Purpose: Evaluation is a systematic process of collecting, analyzing, and interpreting information to determine the extent to which objectives have been achieved. One of the tools or methods for evaluating the students is an open-book examination (OBE). The present study aimed to compare OBEs and closed-book exams (CBEs) and assess students' perception of OBEs.

Methods: A comparative descriptive study was conducted among third-year B.Sc. nursing students (30 OBEs and 30 CBEs) selected using a simple random method on the topic of neonatal resuscitation. Validated knowledge questions with a blueprint on neonatal resuscitation were used, and to assess the perception, a Likert questionnaire was administered.

Results: The study found that OBE students scored a higher mean knowledge than CBE students (13 vs. 10, p<0.05). Students had a positive perception of the OBE (30±5). A total of 78% of students mentioned that they are more comfortable and satisfied with the OBE than the CBE, and they recommended the OBE in their formative assessment, promoting a student-centered approach to education. Additionally, they noted the need to spend more time preparing for the OBE.

Conclusion: The study recommends offering an OBE as an option for formative assessment, in addition to a CBE. As students are encouraged to use classroom notes and textbooks while answering the questions, the “reasoning” rather than recalling the facts could be emphasized. The OBE enhances the learning environment and helps students understand and respond to questions more effectively.

## Introduction

Evaluation in education is a rational act that helps educators assess students' understanding of their learning level [1]. Student evaluation helps assess the teaching and learning process, guiding future teaching actions [2]. One of the tools or methods for evaluating the students is an open-book exam (OBE). In an OBE, students are encouraged to use classroom notes and textbooks while answering the questions. The rationale of such an examination is “reasoning” rather than recalling the facts [3]. OBE enhances the learning environment and helps students understand and respond to questions more effectively [4]. While preparing for OBEs, students consult various sources, including textbooks, classroom notes, and online blogs. This helps them interrelate the concepts and encourages them to acquire knowledge more creatively, thereby avoiding “rote learning” [5]. A closed-book exam (CBE) is a traditional assessment method in which students are not allowed to refer to textbooks, notes, or any other reference materials during the test. It is designed to evaluate a learner’s memory, understanding, and ability to recall information without external aids. CBEs encourage thorough preparation and mastery of the subject matter, fostering critical thinking and application skills. This method is used in academic settings to ensure individual accountability and retention of knowledge. Studies conducted in the past have shown that when comparing students’ scores for OBE and CBE, there is no statistical difference, even though students who took CBE had slightly higher scores [6]. During the coronavirus crisis, the education sector has undergone a transformation. Universities are adopting online lectures, OBEs, online tests, and other digital learning tools [7].

Educational institutions in India are well-acquainted with a traditional face-to-face classroom setup and, therefore, are reluctant to switch to e-learning. Prior to the COVID-19 pandemic, some institutions had gradually started implementing blended learning techniques, but not on a large scale [8]. However, the sudden strike of COVID-19 has forced all the academic units across India to shift to an online teaching mode overnight. In these challenging times, e-learning is a viable solution for addressing the impact of COVID-19 [9]. Academic units should work in a manner that facilitates the introduction of changes more quickly and maintains the quality of education [10]. After looking at the literature, students’ evaluation during the COVID-19 pandemic is an under-theorized topic. Although numerous research pieces are available on how online teaching has become a vital component of the education sector during this pandemic, the impact of this shift on the student evaluation mechanism remains an underexplored topic.

However, as students resumed their regular academic sessions offline after the COVID-19 pandemic, the researcher aimed to assess the difference in performance among nursing students through OBEs and CBEs in offline mode on a selected topic from their curriculum. The present study was undertaken to answer the following questions: Is there any difference in performance among nursing students through OBEs and CBEs? What is the perception of nursing students regarding OBEs?

## Materials and methods

The study employed a quantitative research approach, utilizing a cross-sectional comparative study design, to examine the research objectives. The study was conducted at the College of Nursing, All India Institute of Medical Sciences, Bhubaneswar, India. The study population consisted of nursing students, with the target population specifically being the third-year B.Sc. nursing students. The third-year B.Sc. nursing students were selected as they were exposed to the Neonatal Intensive Care Unit (NICU) environment and curriculum, which covered the Neonatal Resuscitation Program. The study was conducted from March 1 to May 31, 2021. A simple random sampling technique, employing the lottery method, was used to ensure an unbiased selection of participants, thereby enhancing the representativeness and reliability of the study's findings.

Sample size calculation based on the previous study [11] means performance by students with an open book (35.2) and closed book (29.8) using the formula $n = \frac{(Z_{\alpha/2} + Z_{\beta})^{2} \times 2\sigma^{2}}{(\mu_{1} - \mu_{2})^{2}}$. Hence, the calculated sample size was 30 in each group, with a total of 60 students. The study included the students who had undergone Neonatal Resuscitation training and were willing to participate, and excluded students who were on leave during the data collection period. The study included tools and techniques such as a structured questionnaire on “neonatal resuscitation.” It consists of five questions, each worth 5 marks, totaling 25 marks, and is conducted over 40 minutes for both groups (see Appendices). A blueprint for evaluation has been prepared. The perception of students of open books was assessed using the Likert scale. It consists of 10 items on a 3-point rating scale, ranging from "strongly agree" to "strongly disagree," where students were asked to rate their opinion of the OBE. After obtaining ethical clearance and permission from the Principal, all third-year B.Sc. (N) students were enrolled in the Neonatal Resuscitation Training Program. Those 60 students who had undergone neonatal resuscitation training were divided into two groups based on eligibility criteria and consented to participate in two exams: a CBE (30 questions) and an OBE (30 questions) selected by a simple random (lottery) method. After explaining the exam method to both groups, they were seated in two different classrooms. A CBE was administered as a traditional method, and an OBE was conducted by allowing students to use prescribed textbooks in a separate classroom. Using the blueprint of the questions, the answer script was evaluated. Ethical clearance was obtained from the Institutional Ethics Committee, All India Institute of Medical Sciences, Bhubaneswar (T/IM-NF/Nursing/20/95).

Data analysis

The collected data were coded and entered into Microsoft Excel (Microsoft Corp., Redmond, WA, USA) and analyzed using the IBM SPSS Statistics for Windows, Version 25 (Released 2017; IBM Corp., Armonk, New York, United States). Descriptive statistics, including frequency, percentage, mean, and standard deviation, were used to summarize the demographic data and performance scores. Inferential statistics, including the independent t-test, were applied to compare the mean performance scores between the OBE and CBE groups. A p-value of less than 0.05 was considered statistically significant. The perception scores were analyzed using descriptive statistics.

## Results

A total of 60 third-year B.Sc. (N) students participated in the study, with 30 students in each group (OBE and CBE groups). The mean age of the participants was 22 ± 3 years, and all 60 (100%) were female, indicating a homogenous gender distribution within the study sample. The study assessed students’ knowledge performance on neonatal resuscitation through two different examination methods - OBE and CBE. The findings revealed that students who appeared for the OBE achieved a higher mean knowledge score (14 ± 0.6) compared to those who took the CBE (10 ± 0.3). An independent t-test was applied to compare the mean scores between the two groups. The difference was found to be statistically significant (t = -3.72, p = 0.001), indicating that students who participated in the OBE demonstrated significantly better performance in knowledge assessment compared to their counterparts in the CBE (Table 1).

**Table 1:** Comparison of score obtained by students in open-book and closed-book exam Statistical test used: independent t-test; **significance at p-value <0.01.

Method of examination	Mean	t-value	p-value
Open-book exam	14±0.6	-3.72	0.001**
Closed-book exam	10±0.3	-	-

Students’ perception of OBE

Perception toward OBE was assessed using a 10-item Likert scale. The majority of students demonstrated positive attitudes toward the OBE method (Table 2). Most participants (20, 66.6%) agreed that they were comfortable and satisfied with the OBE method, while only two (6.6%) disagreed. A large proportion (21, 70%) felt that OBE improved their understanding of the subject, and 18 (60%) agreed that it encouraged thinking ability. An overwhelming majority (27, 90%) expected to score better through the OBE method, and 28 (93.3%) agreed that OBE reduces the need for memorization. All students (30, 100%) strongly supported the use of OBE in formative assessments, indicating its acceptability and feasibility. Furthermore, 28 (93.3%) participants felt that OBE promotes a student-centered approach to education, and 23 (76.6%) agreed that it requires more preparation time. A significant number (27, 90%) reported that OBE encourages self-directed learning, reflecting its potential in promoting independent study habits. Notably, all students (30, 100%) expressed that OBE reduces examination anxiety, suggesting a psychologically favorable testing environment.

**Table 2:** Frequency and percentage distribution of students’ perceptions toward OBE (n=30) OBE: open-book exam

S. no	Items	Disagree		Neutral		Agree	
		Frequency	%	Frequency	%	Frequency	%
1	I am comfortable and satisfied with OBE	2	6.6	8	26.6	20	66.6
2	OBE method improves understanding of the subject	4	13.3	5	16.6	21	70
3	OBE encourages thinking ability	6	20	6	20	18	60
4	I expect to score better in the OBE method	0	0	3	10	27	90
5	OBE decreases memorizing content	0	0	2	6.6	28	93.3
6	I recommend OBE in formative assessment	0	0	0	0	30	100
7	OBE promotes a student-centered approach to education	2	6.6	0	0	28	93.3
8	I need to spend more time preparing for OBE	1	3.3	6	20	23	76.6
9	OBE encourages self-directed learning in students	1	3.3	2	6.6	27	90
10	OBE reduces my anxiety during exams	0	0	0	0	30	100

## Discussion

The superior performance in the OBE can be attributed to the reduced cognitive load during assessment. OBEs allow students to consult reference materials, which helps alleviate anxiety and enables more focused application of knowledge [12]. This method encourages deeper learning and critical thinking, as students are expected to analyze, evaluate, and synthesize information rather than recall it from memory [13]. In competency-based curricula, such as in medical and nursing education, these attributes are highly valued because they reflect real-world problem-solving and clinical decision-making [14]. Furthermore, the open-book format may promote higher engagement with course materials. When students understand that they need to apply rather than memorize information, they are more likely to study in a meaningful way, focusing on understanding rather than rote learning [15]. Additionally, this approach mirrors authentic professional scenarios where healthcare professionals refer to guidelines, protocols, or evidence-based resources in practice, rather than relying entirely on memory. On the other hand, the CBE, though traditional, appears to disadvantage students in terms of performance. Its emphasis on memory recall may increase stress and test anxiety, both of which can negatively impact exam performance [16].

The findings from this study support emerging pedagogical shifts toward integrating OBEs, especially in higher education and health sciences. According to a recent study, students perceive open-book assessments as fairer and more reflective of actual learning, especially in the post-pandemic academic environment that has embraced hybrid and online modalities [17].

In regard to the opinion of nursing students towards OBE, a striking finding was that all 30 students (100%) unanimously agreed that OBEs reduce examination-related anxiety. Exam anxiety is known to impair student performance, particularly in high-stakes, memory-focused assessments [16]. OBE, by allowing access to learning materials, creates a less pressured environment, promoting calmness and cognitive clarity. Furthermore, all participants (30, 100%) endorsed the use of OBE in formative assessments, signaling strong support for incorporating OBE into ongoing academic evaluations. This reflects a shift from traditional, high-pressure examinations to assessment approaches that support lifelong learning and authentic problem-solving [17].

The results also revealed that 28 (93%) students agreed that OBE reduces the need for memorization, while 27 (90%) indicated that it encourages self-directed learning. These findings support the growing view that OBE fosters higher-order cognitive skills such as analysis, synthesis, and evaluation [13]. When students are not preoccupied with memorization, they are more likely to understand and apply concepts, a crucial skill in professional fields such as nursing, medicine, and allied health sciences.

Additionally, 28 (93%) respondents reported that OBE promotes a student-centered approach, and 27 (90%) agreed that it helps improve subject understanding. These results suggest that OBE is perceived not only as a tool for evaluation but as a catalyst for transforming learning into an active, meaningful process. This aligns with contemporary educational philosophies such as constructivism, which emphasize learning through exploration and context rather than passive content absorption [17].

One of the major strengths of this study is its comparative design, which provides valuable insights into the effectiveness of OBE versus CBE methods in a controlled academic setting. The use of validated knowledge questions with a blueprint ensures the reliability and academic rigor of the assessment tool. Additionally, the inclusion of student perceptions through a structured Likert scale offers a holistic understanding of the cognitive and affective domains involved in learning assessment. The focus on a critical and practical topic, neonatal resuscitation, adds clinical relevance to the findings, especially for nursing students.

However, the study also has limitations; the use of a single topic for assessment may not reflect the broader applicability of OBEs across various subjects or domains. The short-term outcome (knowledge score) does not capture long-term retention or application in clinical practice. Additionally, self-reported perceptions may be subject to bias, such as social desirability bias or the influence of recent test experiences. The topic is practical; however, other topics, such as nursing diagnosis, concept integration mapping, nursing process, and nursing care plans, can also be tested as OBE.

## Conclusions

The findings from this study suggest that OBEs are not only effective in enhancing knowledge acquisition but also positively perceived by students as less stressful and more conducive to critical thinking. Compared to the traditional closed-book format, OBE encourages deeper learning, student engagement, and application of knowledge rather than rote memorization. The favourable perception among students highlights the potential of OBE as a formative assessment tool, especially in competency-based education. Hence, educators should consider integrating OBEs alongside traditional methods to foster a student-centered learning environment and promote reasoning and reflection in academic evaluations.

## Figures and Tables

**Figure 1:**
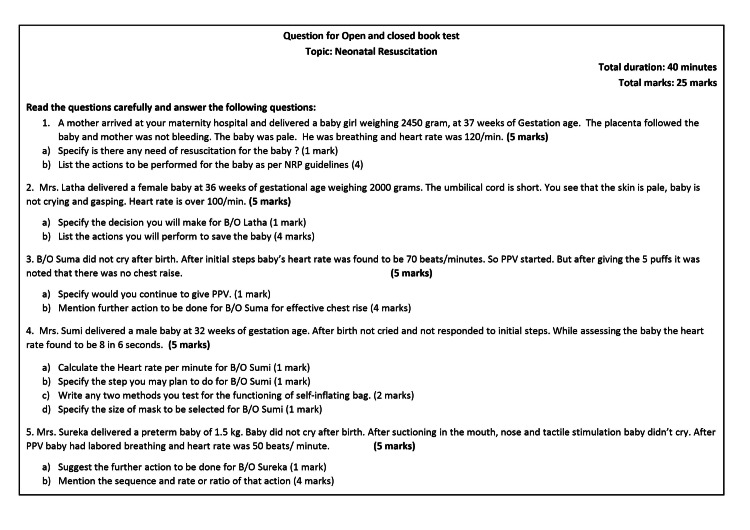
Questions used in open-book and closed-book examinations
